# Integration of Tumor Heterogeneity for Recurrence Prediction in Patients with Esophageal Squamous Cell Cancer

**DOI:** 10.3390/cancers13236084

**Published:** 2021-12-02

**Authors:** Zihang Mai, Qianwen Liu, Xinye Wang, Jiaxin Xie, Jianye Yuan, Jian Zhong, Shuogui Fang, Xiuying Xie, Hong Yang, Jing Wen, Jianhua Fu

**Affiliations:** 1 Department of Thoracic Oncology, Sun Yat-Sen University Cancer Center, Guangzhou 510060, China; maizh1@sysucc.org.cn (Z.M.); liuqw@sysucc.org.cn (Q.L.); wangxy@sysucc.org.cn (X.W.); yuanjy@sysucc.org.cn (J.Y.); zhongjian@sysucc.org.cn (J.Z.); fangsg@sysucc.org.cn (S.F.); yanghong@sysucc.org.cn (H.Y.); 2 State Key Laboratory of Oncology in South China, Collaborative Innovation Center for Cancer Medicine, Guangzhou 510060, China; xiexy@sysucc.org.cn; 3 Guangdong Esophageal Cancer Institute, Guangzhou 510060, China; 4 School of Statistics, Renmin University of China, Beijing 100872, China; xiejx@ruc.edu.cn

**Keywords:** tumor heterogeneity, esophageal cancer, prognosis prediction, cancer cell fraction, mutation

## Abstract

**Simple Summary:**

This manuscript reports a deep sequencing study comprehensively analyzing the clinical impact of mutations considering the abundance of mutations. We built an eight-gene mutation predictor considering intratumoral heterogeneity to predict post-surgery recurrence in ESCC patients. Unlike previous studies that simply treated mutations as binary variables (mutant and wild type), we quantified mutations by the fraction of cancer cells carrying the mutations, and our results showed that the cancer cell fraction of mutations was more informative than the mutation status of genes in recurrence prediction. The predictor was further validated as a powerful recurrence indicator in our validation set and the TCGA-ESCC cohort. With the popularization of targeted deep sequencing in clinical work, our study will help clinicians make accurate predictions of recurrence for patients and will provide a new perspective in the clinical transformation of genomic findings.

**Abstract:**

Esophageal squamous cell carcinoma (ESCC) is one of the deadliest malignancies in China. The prognostic value of mutations, especially those in minor tumor clones, has not been systematically investigated. We conducted targeted deep sequencing to analyze the mutation status and the cancer cell fraction (CCF) of mutations in 201 ESCC patients. Our analysis showed that the prognostic effect of mutations was relevant to the CCF, and it should be considered in prognosis prediction. EP300 was a promising biomarker for overall survival, impairing prognosis in a CCF dose-dependent manner. We constructed a CCF-based predictor using a smooth clipped absolute deviation Cox model in the training set of 143 patients. The 3-year disease-free survival rates were 6.3% (95% CI: 1.6–23.9%), 29.8% (20.9–42.6%) and 70.5% (56.6–87.7%) in high-, intermediate- and low-risk patients, respectively, in the training set. The prognostic accuracy was verified in a validation set of 58 patients and the TCGA-ESCC cohort. The eight-gene model predicted prognosis independent of clinicopathological factors and the combination of our model and pathological staging markedly improved the prognostic accuracy of pathological staging alone. Our study describes a novel recurrence predictor for ESCC patients and provides a new perspective for the clinical translation of genomic findings.

## 1. Introduction

Esophageal cancer is a major cause of cancer-associated death in China [1]. Esophageal squamous cell carcinoma (ESCC), a main pathological subtype of esophageal cancer in China, is characterized by genome instability and poor prognosis. Despite the notable clinical benefit of neoadjuvant therapy, many patients with local advanced ESCC still choose to receive esophagectomy alone because of indisposed health, their economic status, and patient refusal [2]. Therefore, it is important to recognize patients with a high recurrence risk, as surgery alone is not sufficient, and they require intensive treatment. Similar to other solid tumors, the tumor-node-metastasis (TNM) staging system remains the main predictor of the recurrence risk and survival of patients with ESCC; however, patients in the same stage receiving surgery may have quite dissimilar clinical outcomes. Though expedient, this anatomical feature-directed tool is clearly insufficient for the accurate assessment of prognosis, urgently calling for novel prognosis predictors.

In recent decades, the popularization of next-generation sequencing has offered a novel perspective on tumor biology. With the benefit of the vast amount of genomic data, we have gained a glimpse of the prognostic value of a few frequently mutated genes in ESCC, such as NFE2L2 and EP300 [3,4,5,6,7,8]. Assessing patient prognosis on account of the tumor’s genomic background is still far from clinical practice, and more studies are required. To address this issue, we need to consider the intratumoral heterogeneity suggesting that tumors are composed of several clones and that many mutations are carried by minor clones of cancer cells. Genome-/exome-wide sequencing approaches conducted in previous studies have limited power to detect mutations in small clones that might be clinically relevant [9,10,11], so the presence and prognostic impact of many mutations in minor tumor clones in ESCC remain unknown. Moreover, signatures integrating multiple mutational biomarkers have been reported in many cancers [12,13], but few of these promising prognosis predictors have been developed for ESCC. Previous studies treated mutation data as binary variables (mutant or wildtype) in predictor construction [12,13], and the less informative discrete data often yielded unsatisfactory results. Another type of mutation data, the fraction of tumor cells carrying the mutation (CCF), was continuous and might be more suitable in model construction. However, the application of CCF to build recurrence predictors in cancers has not yet been explored.

Recently, targeted deep NGS has entered clinical practice, offering high sensitivity in mutation detection. In the current study, by applying targeted deep sequencing, we aimed to unravel the clinical relevance of frequently mutated genes in ESCC patients, especially those in minor tumor clones. We also made efforts to build a predictor based on cancer cell fractions (CCFs) of mutations and validate the prognostic accuracy of this classifier in a validation cohort.

## 2. Materials and Methods

### 2.1. Sample Selection and Sequencing

All the samples in our study came from surgical specimens and were stored at −80 °C in the biobank at Sun Yat-sen University Cancer Center. Before surgery, patients were checked by CT, PETCT and endoscopy to exclude distant metastasis and staged by experienced oncologists following the AJCC cancer staging manual. In our study, all patients underwent esophagectomy, without receiving neoadjuvant therapy due to their refusal or poor physical conditions. In total, 201 $T_{1-4}N_{1-3}M_{0}$ ESCC patients were identified according to our established selection criteria (See Supplementary Materials, Figure S1). Before performing further experiments, computer-generated random numbers allocated 70% of the patients (N = 143) into the training cohort, and the remaining 58 patients were assigned to the validation set. Among the 201 cases, 48 samples of leukocytes were randomly chosen as normal control. The Ethics Committee of Sun Yat-sen University Cancer Center approved the study design and collection of data and waived the requirement for informed consent given the retrospective nature of the study (SZR2019-109). The study was performed in accordance with the Declaration of Helsinki.

A total of 548 of the most frequently mutated genes were included in our sequence panel based on mutation frequencies. Genomic DNA from frozen tissues was captured by our customized panel (SureSelect, Agilent, Santa Clara, CA, USA) and sequenced using paired-end 150 bp on the Illumina NovaSeq 6000 platform.

### 2.2. Bioinformatic Analysis

The clean reads from both tumor and normal tissues were aligned to human reference genome b37 using BWA [14]. Mutect2 was used to identify variants in 201 ESCC samples [15]. All 48 normal samples were pooled into a normal panel for the filtering of potential germline variants. A bespoke variant selection criterion was developed to distinguish somatic mutations and germline variants in the absence of matched normal sample. (see Supplementary Materials). Copy number analysis was performed using CNVkit, which is well-designed software specific for targeted sequencing [16].

The cancer cell fraction (CCF) of mutation was defined as the fraction of tumor cells carrying a specific mutation and could be calculated using the allele frequency of mutation, copy number of the mutation locus and tumor purity [17]. The clonal status of mutation was inferred by the Bayesian inference [17]. Mutations were classified as clonal on the ground of the probability that the CCF exceed 0.9. A probability threshold of 0.5 was used in our study ($P_{CCF>0.90}>0.5$). We performed stability selection with the package “stabs” to evaluate the performance of models based on the CCFs data (continuous variables) and data of mutation statuses (binary variables).

The predefined training and validation sets were used for model construction and validation. CCFs of mutations per gene per patient were assembled into a matrix (row representing genes and columns samples). A two-step strategy was established to select variables for predictor construction. First, Cox regression with SCAD penalty was applied to select mutated genes associated with disease-free survival. Second, variables were further filtered by stepwise Cox regression with Bayesian information criteria. Patients were stratified by recurrence risk using recursive partition analysis. To minimize the selection bias given the nature of our single-center retrospective study, we further validated our predictor on the TCGA-ESCC cohort (the only cohort that provided omics data and date of disease progress). Other computational methods are detailed in the Supplementary Materials.

### 2.3. Statistical Analysis

No method was used to estimate the sample size. *p* values for survival analysis were calculated from the log-rank test, with all patients followed up for mortality until 31 December 2019. Median follow-up time and 95% confidence interval (CI) were estimated using the reverse Kaplan–Meier method. Student’s *t* test or Wilcoxon rank sum test were used to compare two groups of continuous variables as appropriate. Fisher’s exact test was used to test for association between categorical variables. Area under the ROC curves (AUCs) were compared using Z-test [18]. Two-sided *p* values were considered significant when below 0.05, unless specified. To control false discovery rate, *p* value was adjusted using the Benjamini–Hochberg method.

## 3. Results

### 3.1. Patient Characteristics

The median age of patients was 60 years. No significant differences in pathological stage, surgical approach or number of LNs dissected between training and validation sets were observed (Table 1). About 30% of patients received adjuvant therapy after surgery. The median follow-up time was 49.1 months (95% CI: 44.1–63.0 months), with 135 tumor recurrences during the follow-up period (100 and 35 in the training and validation sets, respectively). The 3-year disease-free survival (DFS) rates were 34.3% in the training set and 39.0% in the validation set. The 3-year overall survival (OS) rates were 42.7% in the training set and 43.3% in the validation set. There was no prognostic difference between the training and validation sets. (Figure S2A,B).

### 3.2. Overview of Genomic Alterations

We performed deep sequencing of 548 genes in the training set and validation set with average fold coverages of target regions at 1097× (range: 617×–1616×) and 1036× (range: 768×–1445×), respectively. The sequencing depth of the normal sample was 1012× (range: 499×–1519×). In total, more than 95% of targets were covered by 100 reads in both cohorts. Both datasets shared a similar distribution over the allele fraction of mutations (Figure S2C). We combined both datasets to enlarge the sample size in order to analyze the clinical relevance of mutations.

**Figure 1:**
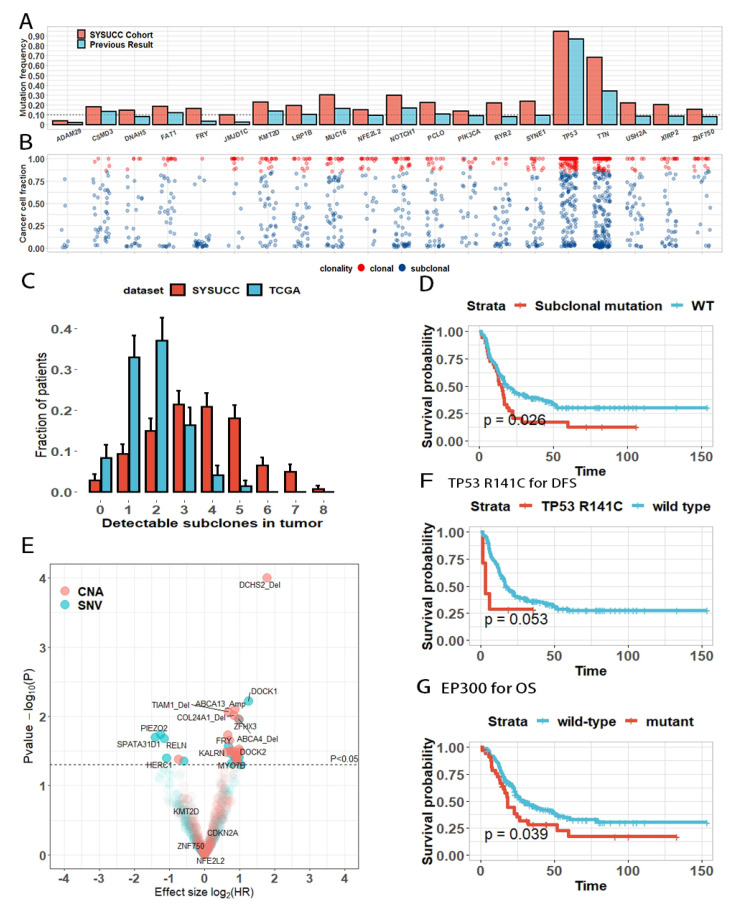
Heterogeneity and clinical impact of alterations in ESCC. (**A**) Bar plot for comparison of mutation frequencies of the most frequently mutated genes observed in previous results and our cohort. (**B**) Scatter plot of cancer cell fraction of mutations in these frequently mutated genes. Clonal mutations are shown in red and subclonal mutations in blue. (**C**) Predicted number of subclones in ESCC. For comparison, the predicted number of subclones from patients in TCGA-ESCC cohort is also shown. Error bar represents the standard deviation. (**D**) Disease-free survival difference between patients with subclonal FRY mutations and wildtype FRY. (**E**) Volcano plot displays the relationship between genetic alterations and DFS. The X and Y axes indicate $log_{2}^{HR}$ and $-log_{10}^{P}$, respectively. “Amp” and “Del” represent the amplification and deletion of the gene, respectively. (**F**) TP53 R141C hotspot mutation was associated with inferior DFS. (**G**) EP300 mutation was associated with grave OS.

Across the entire dataset, 8865 somatic mutations were identified, of which 519 mutations were short insertions or deletions. We selected 56 mutations for further validation and 55 (98.2%) of the selected mutations were confirmed by Sanger sequencing (Figure S9, Table S2). Among the 8865 mutations, 6343 were subclonal mutations observed in 96.5% of patients (194 of 201). Since our deep sequencing approach detected massive mutations in low abundance, the mutation frequency for most genes was higher than that in previous studies (Figure 1A,B). Following the statistical frameworks described previously [19,20], we calculated the CCFs of mutations, inferred their clone status and assigned them into tumor cell subpopulations using “DPclust”. Focusing on 140 samples with tumor purity ≥70%, we observed that many patients owned at least two detectable subclones in addition to a major clone, confirming the huge tumor heterogeneity under the treatment-naive state (Figure 1C). For comparison, we analyzed the whole-exome sequencing data of ESCC in the TCGA cohort [21] and found that the proportion of patients that harbored more than two subclones was higher than that in TCGA cohort (88% vs. 59%, Fisher’s exact test, *p* < 0.001). The predicted number of subclones was larger in the later stage of diseases and higher sequencing depth when we limited the analysis to patients with similar stages and sequencing depth (Figure S2D,E).

We further questioned if mutations in some genes tend to be clonal or subclonal. Using a binomial model, we identified some mutational preferences (Table S7). We did not observe any gene mutated exclusively in a clonal manner. An ESCC-associated gene, ADAM29 [8], was mutated in an exclusively subclonal manner (8/8), including a mutational hotspot, 4 ADAM29 p.Q805 frame shift deletion, although this observation did not reach statistical significance (Figure 1B, *p* = 0.11). FRY was usually mutated in a subclonal manner (35/36, FDR = 0.006), detectable on average in 11% of the tumor cells in mutant patients (Figure 1B). FRY mutations in subclones might endow tumor survival advantages and drive recurrence, evidenced by the shorter DFS of the subclonal mutant compared with that of wildtype patients (Figure 1D).

### 3.3. Clinical Relevance of Genetic Alterations

Globally, we found that lower tumor mutation burden (TMB) was significantly associated with the patient’s drinking status (*p* = 0.012), in addition to smoking status and gender (Figure S3A). At the gene level, we observed that some ESCC mutational drivers were enriched in smokers (EP300) and drinkers after multiple test corrections (Table S3, FAT1 and ADAM49, FDR < 0.05) [8].

We further conducted univariable Cox regression with SNVs and gene-level CNAs as categorical variables. We only included those alterations with frequencies ≥5%, and identified 12 mutations and 19 gene-level CNAs that were associated with disease recurrence (Figure 1E, Table S4). Patients with mutations in the DOCK family (DOCK1 and DOCK2), which are crucial regulators of tumor and leukocyte migration, had worse DFS (Cox regression, DOCK1 hazard ratio (HR): 2.39 (1.29–4.45), *p* = 0.006; DOCK2 HR: 1.98 (1.04–3.77), *p* = 0.041, Figure S4). After correction for multiple comparisons, only DCHS2 deletion remained significantly associated with inferior DFS (HR: 3.43 (1.84–6.40), FDR = 0.043, Figure S4). When we focused on hotspot mutations, we found that patients with the hotspot mutation TP53 R141C (7/201) appeared to have unfavorable DFS (HR: 2.58 (0.97–5.77), *p* = 0.06), but this observation failed to reach statistical significance probably because of the limited sample size (Figure 1F). We confirmed that the EP300 mutation was associated with inferior OS (HR: 1.58 (1.02–2.46), *p* = 0.041) [4,7] (Figure 1G, Table S5). After correction for multiple comparisons, no single mutation or gene-level CNA was significantly associated with OS.

### 3.4. The Prognosis Effect of Mutations Is Related to Cancer Cell Fraction

In the process of analyzing the clinical relevance of mutations, we noticed that patients were with different prognoses even if they carried the same mutated gene. From the genomic perspective, this phenomenon could be explained by different cancer cell fractions (CCFs) of mutations [9,10,11].

**Figure 2:**
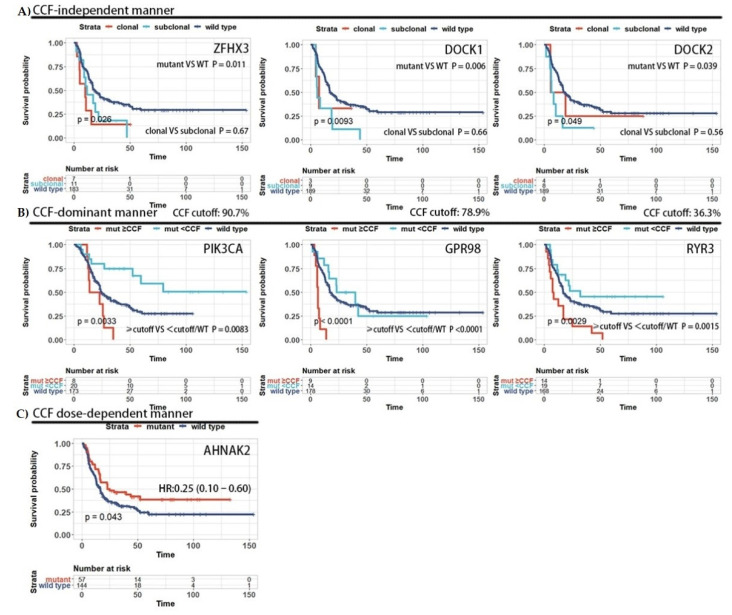
CCF–based patterns of prognostic value. The clinical endpoint analyzed here was DFS. Three prognostic effect patterns were identified by on our classification pipeline: CCF-independent pattern (**A**), CCF–dominant pattern (**B**) and CCF dose–dependent pattern (**C**).

The CCF of a mutation, describing the proportion of tumor cells carrying a mutation, may well represent intratumoral heterogeneity. To determine whether and how the CCF of mutations affected the clinical outcome of patients, we analyzed the prognostic effect of the CCF of mutated genes by an algorithm based on the maximal selected rank statistics, log-rank test and Cox regression (Figure S8). We identified different effect patterns of mutated genes: (1) For the CCF-independent pattern, the prognostic effects of mutations were independent of the CCFs (ZFHX3 and DOCK1 for DFS; ZFHX3 and FAT1 for OS; Figure 2A and Figure S5A). (2) For the CCF-dominant pattern, the mutations exerted their prognostic effects when the CCFs exceeded a threshold (GPR98 and PIK3CA for DFS; GPR98, LAMA5 and FLG for OS; Figure 2B and Figure S5B). (3) For the CCF dose-dependent pattern, the more tumor cells carried given mutations, the more notably the prognostic effect of the mutations (AHNAK2 for DFS; EP300 for OS; Figure 2C and Figure S5C). A detailed classification of the prognostic influence of each gene is listed in Table S6. Note that this analysis pipeline contains multiple statistical tests that were unsuitable for multiple test corrections.

Taking the canonical oncogene PIK3CA as an example, several studies reported that PIK3CA mutations were associated with favorable prognosis [5,6], but some studies provided contradictory results [22]. In our study, PIK3CA mutations had a marginal positive effect on DFS (log rank test, *p* = 0.07) when only considering mutation status (Figure S4A). Considering the abundance of mutations, in terms of CCF, we observed distinct clinical outcomes in patients with different CCFs of PIK3CA mutations, and mutations with CCF greater than 90.7% were associated with reduced DFS and OS (Figure 2B). These results support our hypothesis that the prognosis effect of mutations is relevant to CCF.

### 3.5. Construction of a Recurrence Predictor Using Sequencing Data

As the CCFs of mutations may result in better stability of variable selection and higher prediction accuracy (Figure S6), we constructed a recurrence predictor based on the CCFs of mutations. In the training cohort, we applied SCAD in the Cox proportional hazards context with 10-fold cross-validation and further selected independent prognostic variables using stepwise regression with Bayesian information criterion. Ultimately, eight genes, namely, GPR98, LAMA1, IFT140, MUC17, PTPRB, AHNAK2, PREX2 and STAPA31D1, were included in the genetic model. Moreover, we repeated the random generation of training and validation sets, and iteratively performed variable selection to calculate the probabilities of these eight genes being selected into the model. We found that five genes were selected with probabilities up to 40%, indicating the robustness of our model (Table S8). After the test of proportional assumption (*p* = 0.39), a formula was generated using a Cox regression model to calculate the recurrence risk score in ESCC patients in the training set based on the CCFs of mutated genes:

(1) $$\begin{array}{ll} \mathrm{risk}\;\mathrm{score}=1.18 & \times CCF_{GPR98}+1.31\times CCF_{LAMA1}+1.42\times CCF_{IFT140}\\ & +1.18\times CCF_{MUC17}+1.78\times CCF_{PTPRB}\\ & -1.37\times CCF_{AHNAK2}-2.78\times CCF_{PREX2}\\ & -3.02\times CCF_{SPATA31D1} \end{array}$$

### 3.6. Prognostic Value of the Eight-Gene Classifier

**Figure 3:**
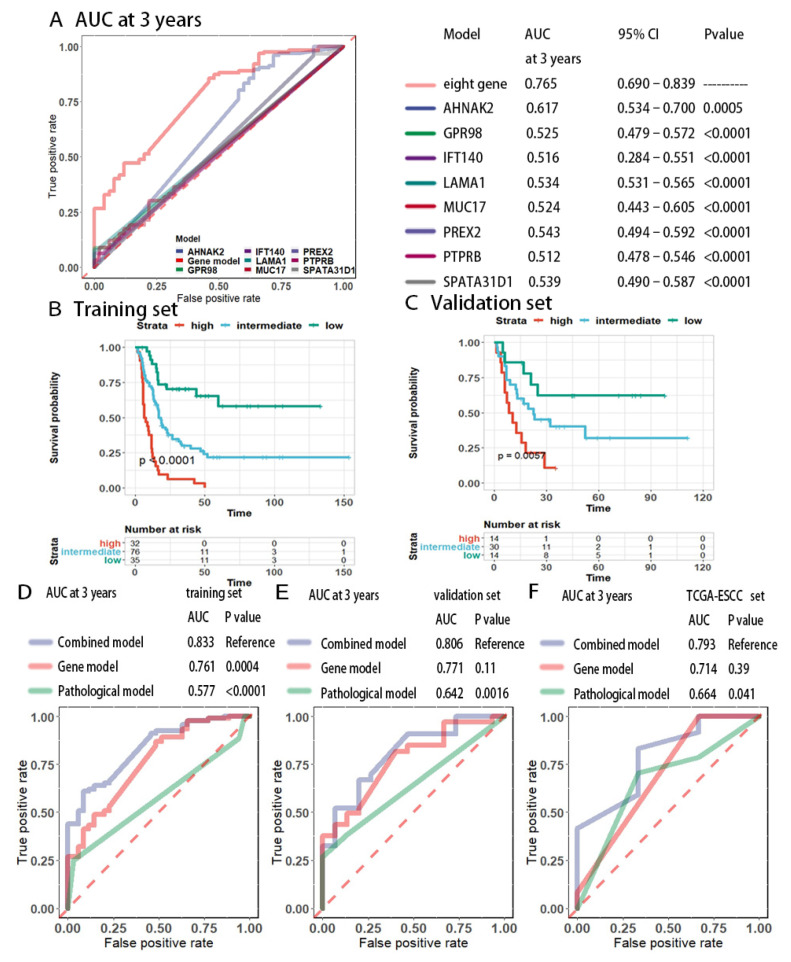
Performance of the eight-gene model in the training and validation sets. (**A**) AUC of the time–dependent ROC curve for the eight–gene predictor and the individual genes across the entire cohort. (**B**,**C**) Patients stratified by risk scores had distinct DFS in the training set (**B**) and validation set (**C**). (**D**–**F**) Time–dependent ROC curves compared the prognostic accuracies of the combined model integrating the eight–gene signature and pathological staging with pathological staging in the (**D**) training cohort, (**E**) validation cohort and TCGA–ESCC cohort (**F**).

The median risk score of 201 patients was 0 (IQR: −0.024to 1.122). In the entire set, the area under the receiver operating characteristic curve (AUROC) of the classifier at 3 years was 0.765, and the AUC of the eight genes at 3 years ranged from 0.512 to 0.617. The eight-gene predictor exhibited a larger AUROC than that of any single gene alone (Figure 3A).

We performed recursive partition analysis to define the optimal cutoff value of the risk score in the training cohort; the resulting cutoffs were −0.0565 and 0.168. Using the cutoff values, patients were stratified into three groups with distinct recurrence risks. The 3-year DFS rate was 6.3% (95% CI: 1.6–23.9%) for the high-risk group, 29.8% (95% CI: 20.9–42.6%) for the intermediate-risk group, and 70.5% (95% CI: 56.6–87.7%) for the low-risk group (Figure 3B). The stratification power was verified in the validation set (*p* = 0.0057). In the validation set, the 3-year DFS rate was 10.7% (95% CI: 1.9–39.3%) for the high-risk group, 40.2% (95% CI: 25.2–64.0%) for the intermediate-risk group, and 62.9% (95% CI: 44.9–94.9%) for the low-risk group (Figure 3C). Although the eight-gene model was built to predict DFS, the three groups of patients still showed distinct OS patterns, indicating the robustness of our predictor (Figure S7A,B).

Finally, we performed a multivariable analysis to determine whether the eight-gene signature was an independent prognostic factor in different populations. As summarized in Table 2, the eight-gene signature remained an independent prognostic indicator after adjustment for clinicopathological factors in both the training set and validation set. Time-dependent ROC analysis demonstrated that, in contrast to the pathological model, the combined model integrating the eight-gene mutation signature with pathological staging achieved a remarkable increase in prediction performance in both the training set (AUC = 0.833, *p* < 0.0001) and validation set (AUC = 0.806, *p* = 0.0016, Figure 3D,E). We further validated our CCF-based predictor in the TCGA-ESCC cohort. In accordance with the results of our cohort, the eight-gene mutation signature achieved better performance than that of the AJCC7th pathological stage, and the combined model had a higher AUC, suggesting that the eight-gene-based predictor was a powerful predictor adding prognostic information to canonical pathological staging (AUC = 0.793, *p* = 0.041, Figure 3F).

### 3.7. Specific Genotype of Long Survivors

**Figure 4:**
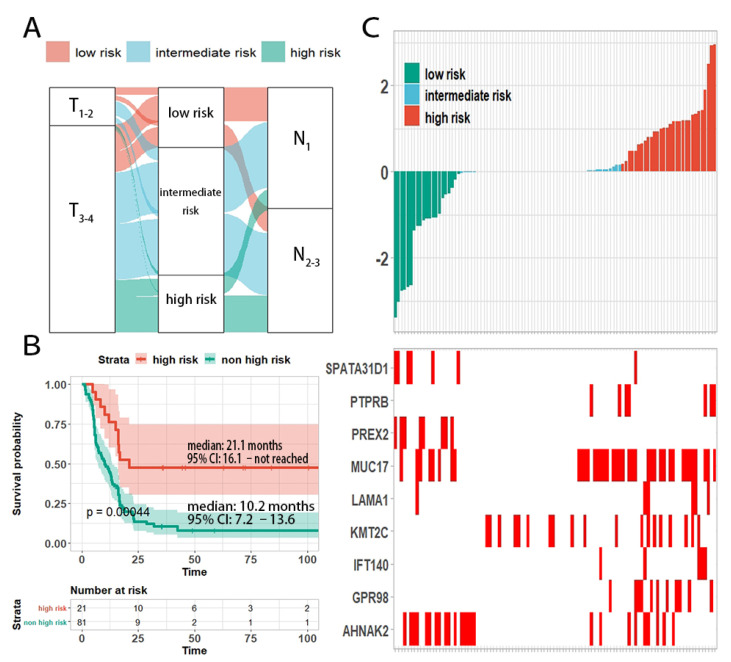
Risk stratification of patients with $N_{2–3}$ staging. (**A**) Sankey plot displaying the relationship between the risk scores of patients and different pathological stages. (**B**) Survival curves showing that patients with $N_{2–3}$ but low genetic risks had a relatively longer DFS time. (**C**) Heatmap showing the mutation profile of $N_{2–3}$ ESCC patients in different recurrence risk. PREX2 and SPATA31D1 mutations were enriched in $N_{2–3}\;$ low-risk patients.

When we compared the differences of prognosis stratifications of TNM stage and our genetic predictor (Figure 4A), we noticed that 20.6% (21/102) of $N_{2–3}$ cases were classified as low risk according to our genetic classifier. These pathological-stage-defined high recurrence risk patients had a relatively longer DFS time than that of other $N_{2-3}$ patients (Figure 4B), indicating lower aggressiveness of these tumors. The tumors of these long-surviving $N_{2–3}$ patients were enriched for PREX2 mutations (38.1% (8/21) vs. 0% (0/81)) and SPATA31D1 mutations (28.6% (6/21) vs. 1.2% (1/81)). Importantly, all these patients displayed the KMT2C wildtype (0% (0/21) vs. 23.5% (19/81)), which is a known tumor suppressor in ESCC [4,8] (Figure 4C). Similarly, 17.2% (17/99) of $N_{1}$ cases were stratified into the high-risk group, and they experienced more rapid recurrence (Figure S7C). However, no mutations were enriched in this subset of patients. As the TNM staging system reflected the natural course of ESCC, our prediction model evaluated the invasiveness of ESCC from the genomic perspective.

## 4. Discussion

In this study, we performed targeted deep sequencing on the most significantly mutated gene loci of 201 patients to gain in-depth insight into the clinical relevance of frequently mutated genes in ESCC. We found that drinking status was associated with TMB and frequent FAT1 and ADAM29 mutations, implying that lifestyle factors might shape different mutational landscapes and the tumor biology of ESCC [7]. The clinical impact of some mutations has been described previously, and we extended the study to systematically investigate the associations between frequent mutations, gene-level CNAs and patient prognosis. In accordance with previous reports [4,7,23,24], our analysis demonstrated that FAT1, ZFHX3 and EP300 mutations were associated with unfavorable OS in ESCC. In contrast to a previous study [3], we failed to confirm the prognostic effect of NFE2L2 and CSMD3 [3,25] (Figure S4G,H), probably due to differences in patient pathological stages, because all of the patients recruited in our study had LN metastasis. Note that the insufficient coverage of our sequencing panel, which prevented us from analyzing the clinical relevance of broad CNAs, might be a limitation of our study.

Previous genome-wide (WGS/WES) studies had inadequate power to detect mutations in very small clones that would expand over time and impact patient outcome [9,10,11,17]. By applying deep sequencing, we stated that the prognostic impact of some mutations could be quantified by the fraction of cancer cells carrying a given mutation (namely, the CCF). Among these mutations, EP300 mutation was a promising biomarker of dismal OS [4,7], which might impair patient OS in a dose-dependent manner. Additionally, some mutations affected prognosis of patients independent of their CCF, suggesting that clonal mutations had similar impacts on prognosis with mutations in minor clones. This additional information on small clones detected by deep sequencing could allow broader identification of patients of unfavorable prognosis and minor clones of biological and prognostic value.

Another important issue we attempted to address is the translation of genomics features into prognosis assessment for patients. Our study took advantage of targeted DNA sequencing, an extensively used tool in clinical practice, to make our findings directly clinically relevant. Recently, some studies successfully built prognosis predictors using genomic data [12,13]. These previous models simply considered the mutation status as a binary variable, ignoring tremendous intratumoral heterogeneity. As the CCF of mutations was more informative in the impact on prognosis, we developed and validated a powerful model based on the CCFs of mutations of eight genes to predict recurrence in ESCC patients. The variable selection method that we used was the SCAD model, which can provide unbiased estimation of coefficients of variables and is theoretically better than the least absolute shrinkage and selection operator (LASSO) model [26]. As an independent prognostic factor, the eight-gene signature exerted prediction performance that was better than that of canonical pathological staging. Furthermore, complementation of pathological staging with the genetic model could result in a notable increase in predictive performance. We also assessed the predictive capacity of our eight-gene signature in the TCGA-ESCC cohort, and our predictor showed robust performance, although the sequencing depth of the TCGA-ESCC cohort was extremely lower than that of our datasets (on average 55× in the TCGA dataset and 1000× in our datasets), which might have led to lower detection rates of somatic mutations.

The association between the genetic model and ESCC recurrence can be explained by the biological function of genes. Overexpression of LAMA1 promotes ESCC proliferation [27]. IFT140, is required for cell motility, and its promoter hypermethylation serves as a risk factor for pancreatic cancer [28]. MUC17 is reported to be a tumor suppressor in gastric cancer [29]. PTPRB is a negative regulator of angiogenesis and is frequently mutated in angiosarcoma [30]. AHNAK2 is associated with tumorigenesis of renal cancer [31]. As a frequently mutated gene in multiple cancers [32], PREX2 can accelerate tumor proliferation and invasion [33]. To the best of our knowledge, the function of SPATA31D1 in cancer remains unknown. Previous studies have identified several driver mutations in ESCC, but the gene lists are still incomplete because of the limited sample sizes [34]. Although none of the eight genes were previously known as ESCC drivers, we observed some hotspot mutations, suggesting that these genes underwent positive selection pressure (Figure S10). Therefore, the functional consequences of mutations in these genes in ESCC deserve further research.

Some patients with extensive LN metastasis still had a relatively longer survival time and they all had wildtype KMT2C. Coincidentally, Hao reported that all 20 long survivors (OS > 3 years) with metastatic gastroesophageal adenocarcinoma (stage IVb) had wildtype KMT2C [35]. Additionally, KMT2C decreased the expressions of EMT-related genes and cancer cell migration [35,36]. Further studies are required to address whether this key component of DNA methylation shapes different tumor biologies in ESCC.

## 5. Conclusions

In summary, our results provided the first report to systematically analyze the clinical impact of mutations while comprehensively considering their clone sizes in ESCC. We integrated the information of intratumoral heterogeneity into model construction and built a reliable recurrence predictor based on the CCFs of mutations in eight genes, affording additional prognostic value to the standard TNM stage system. The popularization of cost-effective panel sequencing will further realize the potential of our findings in understanding clinical heterogeneity and assessing patient prognosis.

## Figures and Tables

**Table 1:** Main clinical characteristics of patients in the training and validation cohorts.

Variables	Training Set (N = 143)	Validation Set (N = 58)		Full Set (N = 201)
	N (%)	N (%)	*p*	N (%)
Sex			0.002	
Female/Male	17/126 (11.9/88.1)	18/40 (31.0/69)		35/166 (17.4/82.6)
${\mathrm{Age}}^{\;§}$			0.206	
<60/≥60	70/73 (49.0/51.0)	22/36 (37.9/62.1)		92/109 (45.8/54.2)
${\mathrm{Tumor}\;\mathrm{length}}^{\;§}$			0.222	
<4 cm/≥4 cm	67/76 (46.9/53.1)	21/37 (36.2/63.8)		88/113 (43.8/56.2)
Smoking status			0.063	
Yes/No	98/45 (68.5/31.5)	31/27 (53.5/46.5)		129/72 (64.2/35.8)
Alcoholism			0.520	
Yes/No	73/70 (51.0/49.0)	26/32 (44.8/55.2)		99/102 (49.3/50.7)
Differentiation			0.343	
Well/Moderate/Poor	17/71/55 (11.9/49.7/38.5)	8/34/16 (13.8/58.6/27.6)		25/105/71 (12.4/52.2/35.3)
Surgical approach			0.609	
Left thoracotomy	49(34.3)	17(29.3)		66(32.8)
Right thoracotomy	94(65.7)	41(70.7)		135(67.2)
Lesion location			0.151	
Upper/Middle/Lower	8/54/51 (5.6/58.7/35.7)	8/31/19 (13.8/53.4/32.8)		16/115/70 (7.9/57.2/34.9)
${\mathrm{pT}\;\mathrm{classification}}^{\;†}$			0.848	
$T_{1-2}$ $/T_{3-4a}$	23/120(16.1/83.9)	8/50(13.7/86.3)		31/170 (15.4/84.6)
${\mathrm{pN}\;\mathrm{classification}}^{\;‡}$			1.000	
$N_{1}$ $/N_{2-3}$	70/73 (49.0/51.0)	29/29 (50.0/50.0)		99/102(49.3/20.7)
${\mathrm{LNs}\;\mathrm{examined}}^{\;§}$			0.696	
≥21/<21	108/35 (75.5/24.5)	46/12 (79.3/20.7)		154/47 (76.4/23.4)
Adjuvant therapy			1.00	
Yes/No	43/100 (30.1/69.9)	17/41 (29.3/70.7)		60/141 (29.8/69.2)

†: pathological T classification. ‡: pathological LN classification. §: stratified by median of variables.

**Table 2:** Multivariate Cox regression of the eight-gene-based predictor and clinicopathological factors. †: pathological T classification. ‡: pathological LN classification. §: stratified by median of variables.

Variables	Entire Cohort		Training Cohort		Validation Cohort	
	HR (95%CI)	*p*	HR (95%CI)	*p*	HR (95%CI)	*p*
Sex (male vs. female)	0.45 (0.26–0.77)	0.004	1.18 (0.61–2.31)	0.625	0.13 (0.05–0.38)	0.0001
$\mathrm{Age}{}^{§}$(≥60 vs. <60)	0.75 (0.52–1.09)	0.130	0.67 (0.44–1.03)	0.071	0.45 (0.20–0.99)	0.046
Surgical approach (right vs. left thoracotomy)	0.72 (0.50–1.05)	0.102	0.52 (0.33–0.82)	0.005	1.17 (0.54–2.56)	0.688
Alb (≥40 vs. <40)	0.46 (0.11–2.01)	0.304	1.86 (0.24–14.18)	0.548	0.30 (0.033–2.56)	0.294
$\mathrm{LNs}\;\mathrm{examined}{}^{§}$ (≥21 vs. <21)	1.19 (0.83–1.71)	0.351	0.99 (0.64–1.53)	0.952	1.51 (0.74–3.08)	0.262
$\mathrm{pT}\;\mathrm{classification}{}^{†}$ $(T_{3-4a}\;$$\mathrm{vs}.\;T_{1-2}$)	1.03 (0.60–1.77)	0.902	1.31 (0.69–2.50)	0.408	0.43 (0.15–1.28)	0.128
$\mathrm{pN}\;\mathrm{classification}{}^{‡}$ $(N_{2-3}$$\;\mathrm{vs}.\;N_{1}$)	2.37 (1.63–3.44)	<0.001	2.71 (1.73–4.25)	<0.001	2.29 (1.07–4.92)	0.033
Genetic model (low risk)	-	-	-	-	-	-
Intermediate risk	2.74 (1.59–4.72)	<0.001	2.65 (1.40–5.03)	0.003	3.52 (1.23–10.10)	0.019
High risk	6.50 (3.60–11.75)	<0.001	6.74 (3.34–13.58)	<0.001	8.19 (2.484–27.01)	<0.001

## Data Availability

FASTQ files will be uploaded to Genome Sequence Archive in BIG Data Center (https://bigd.big.ac.cn/gsa/, accessed on 4 October 2021), with the accession code HRA000777. The study’s authenticity has been validated by uploading the key raw data onto the research data deposit (RDD) public platform (https://www.researchdata.org.cn, accessed on 4 October 2021, approval RDD number: RDDB2021001598).

## Supplementary Materials

The following are available online at https://www.mdpi.com/article/10.3390/cancers13236084/s1. Figure S1 Diagram of sample selection; Figure S2 Comparison between training cohort and validation cohort; Figure S3 Clinical relevance of genetic features; Figure S4 Survival curves of patients grouped by mutation status of genes; Figure S5 CCF-based patterns of prognostic value; Figure S6 Variable selection based on CCF and mutation status data; Figure S7 Kaplan-Meier Curves for OS of patients with distinct recurrence risks; Figure S8 Classification pipeline for distinguishing prognosis effect patterns of mutations; Figure S9 Examples of Sanger sequencing validation; Figure S10 Lillipop figure displaying distribution of mutations of the eight genes included in the recurrence predictors; Table S1 Summarization of studies used for designing our gene panel. EAC and ESCC are the abbreviation of esophageal adenocarcinoma and squamous cell carcinoma, Table S2 Sanger sequencing, Table S3 Clinical variables associated with gene mutations. *p* value are calculated with Fisher’s exact test with Bonferroni-Holm correction for multiple comparison, Table S4 Univariable cox regression analysis between disease-free survival and genetic alteration(SNVs and CNAs), Table S5 Univariable cox regression analysis between overall survival and genetic alteration(SNVs and CNAs), Table S6 CCF-based prognostic effect pattern of mutations, Table S7 Binomial test of preference of clone status of oncogenic mutation, Table S8 Probability of the right genes being selected into the CCF-based predictor during 1000 simulations, Table S9 gene panel used in our study (N = 548).
