# Supplementary material for: Integration of Tumor Heterogeneity for Recurrence Prediction in Patients with Esophageal Squamous Cell Cancer
Source: Cancers (Basel). 2021 Dec 2;13(23):6084. doi: 10.3390/cancers13236084 (PMC8656931; doi:10.3390/cancers13236084)
Supplement: Supplementary file 1 [file cancers-13-06084-s001.zip › supplementary_note_targeted_sequencing.pdf]

CATALOGUE

Supplementary methods 1
Sample selection 1
Determinant of tumor purity 2
Treatment, follow up of patients and collection of clinical data 2
Gene panel design and sequencing 3
Reads alignment and Variant calling 3
Copy number analysis 4
Sanger sequencing 4
Estimation of cancer cell fraction and inferrence of clone status 4
Classification pipeline for CCF-based pattern with influence on prognosis 5
Comparison of model construction using CCF data and genotype data 5
Model construction and evaluation 6
Clustering analysis of mutations using Bayesian Dirichlet Process 7
Supplementary figure 7
Figure S1 Diagram of sample selection 8
Figure S2 Comparison between training cohort and validation cohort 8
Figure S3 Clinical relevance of genetic features 9
Figure S4 Survival curves of patients grouped by mutation status of genes 9
Figure S5 CCF-based patterns of prognostic value 10
Figure S6 Variable selection based on CCF and mutation status data 10
Figure S7 Kaplan-Meier Curves for OS of patients with distinct recurrence risks 11
Figure S8 Classification pipeline for distinguishing prognosis effect patterns of mutations 12
Figure S9 Examples of Sanger sequencing validation 13
Figure S10 Lillipop figure displaying distribution of mutations of the eight genes included in the recurrence predictors. 13

SUPPLEMENTARY METHODS

Sample selection

Our study was conducted on 201 ESCC surgical samples (fresh frozen tissues). All the samples were derived from esophagectomy and stored at -80 °C for long-term preservation in the biobank of Sun-Yat Sen University Cancer Center. All patients underwent esophagectomy, achieved complete resection without receiving neoadjuvant therapy and experienced pathological-validated lymph node metastasis. Samples were chosen following our established criteria (Figure S1).

Inclusion criteria contained:

(1) Patients whose age ≥18 years old, preoperative KPS score ≥90;

(2) Pathological diagnosis as esophageal squamous cell carcinoma and tumor located in thoracic segment of the esophagus;

(3) R_0 resection via thoracic approach and standard lymph node excision;

(4) Neo-adjuvant treatment naïve;

(5) Patients experienced lymph node metastasis confirmed by pathological diagnosis

Exclusion criteria contained:

(1) Patients with secondary primary tumor;

(2) Patients with distant metastasis found by PET-CT.

(3) Patients died within 30 days after surgery or died of post-operation complication;

(4) Patients lacking essential clinical information, such as age, sex, operation record, pathological diagnosis and follow-up data.

Determinant of tumor purity

All samples underwent pathological review via frozen section. A tissue section was created with two H&E slides (termed as top and bottom): a 4 μm frozen section (top slide) was cut, 20 mg of tumor tissue was shaved from the tissue for library construction, then a second 4 μm frozen section was cut (bottom slide). An H&E stain was conducted on both slide tissue sections. SYSUCC-authenticated pathologist conducted diagnosis verification and tumor purity assessment. Pathologist initially screened the slide in low magnification to determine the microscopic morphology, then magnified to 20X and reviewed 10 representativefields on each slide. The tumor purity was derived from the proportion of tumor nuclei compared to the total nuclei present on the slide. The tumor purity of each sample was the average level of purities in both top and bottom slides. For quality control, a random review of 20% of slides was conducted by a second pathologist to confirm the results. If the results of the second review were off by 10%, the sample would be assessed again.

Treatment, follow up of patients and collection of clinical data

Before surgery, patients were systemically assessed by CT of the neck, chesk and upper abdomen, endoscopy, and PETCT for accurate staging. They were staged by experienced oncologists following the AJCC cancer staging manual and underwent esophagectomy, without receiving neoadjuvant therapy due to patients' refusal or poor physical condition. The surgical procedures included right-sided transthoracic approach (McKeown or Ivor-Lewis) and left-sided transthoracic approach (Sweet), with minimally invasive or open techniques. As the guidelines recommended, patients with pathological-validated lymph node metastasis received adjuvant chemotherapy, radiochemotherapy or surveillance according to patients' choice. Patients were followed up through regular outpatient service four times per year within the first year after surgery, twice per year from the second to the fifth year, and once a year after the fifth year. Regular examination included physical examination, routine blood and biochemical examination, tumor biomarkers (SCC and CEA), endoscopy and CT. Patients were given chemotherapy or

radiochemotherapy following the clinician's recommendations if they experienced disease relapse. Demographic and clinical data were extracted from our clinical database.

Clinical endpoint data was prepared following the commonly used criteria[1]. Disease free survival (DFS) is defined as the period from the date of surgery to the date of the first tumor recurrence event with radiological or pathological confirmation. The censored time is from the date of surgery to the last contact date or date of death. Overall survival (OS) is the period from the date of surgery until the date of death at any cause. The censored time is from the date of surgery to the date of the last contact. Comprehensive pathological staging was conducted by experienced clinicians following the 8th edition of the AJCC cancer staging manual.

Gene panel design and sequencing

Mutation data were downloaded from supplementary materials of published results[2-9]. Major information of these studies was summarized in Table S1. Then we calculated mutation frequency for each gene based on 589 WGS/WES data. We brought genes with mutation frequency above 2% into our panel list. Ultimately, all exons of 548 selected genes covering 5.731 Mbp were used to design complementary probes for library construction (Table S9).

AllPrep DNA Universal Kit was used to extract DNA from frozen fresh tissues (purity>50%, median: 70%). DNA was quantified and quality controlled by Qubit 2.0 and Agarose gel electrophoresis assay prior to library construction. DNA was broken into 180-280 bp and all exons of 548 genes were captured using Agilent SureSelect XT Custom Kit. After PCR amplification and quality control, the DNA library was sequenced using paired-end 150 bp on Illumina Novaseq 6000 platform.

Reads alignment and Variant calling

Clean reads were obtained after filtering out low-quality reads and adapters from raw reads of both the tumor and normal samples. The clean reads were aligned to human reference genome b37 using BWA[10] and deduplicated using SAMBAMBA[11]. Mutect2 was used to identify variants in 201 ESCC samples[12]. All 48 normal samples were pooled into a normal panel for filtering potential germline variants. All variants were annotated using ANNOVAR[13]. To account for the absence of matched control, a custom variant sifting pipeline was developed, using criteria similar to previous studies[14-16]:

(1) Removal of variants located within low-coverage (<10X) regions and variants with less than 5 mutant reads.
(2) Removal of variants whose allele fraction is 1
(3) Removal of variants with synonymous amino acid alterations on all transcript corresponding to each gene.
(4) For variants with well-characterized annotation in COSMIC[17], removal of known polymorphisms reported among 1000 Genome, Exome Aggregation Consortium data[18] or in-house database at a frequency above 0.1

(5) For variants without annotation in COSMIC, removal of variants recorded in dbSNP, variants with a frequency above 0.003 in 1000 Genome data, variants with a frequency above 0.01 in in-house database and variants with frequency above 0.001 in Exome Aggregation Consortium data[18]
(6) Removal of germline variants present in any of normal panel.

After filtering the probable germline variants, the remaining mutations were used for further analysis in our study.

Copy number analysis

Copy number alterations were identified using CNVkit[19] which was designed specific for targeted sequencing data. In brief, the read counts of 48 normal samples were normalized and integrated into a pool reference. Then targeted reads and nonspecifically captured off-target reads from tumor samples were used to infer somatic copy number alterations. The algorithm also adjusted the bias that led to sequencing read depth: GC content, target size, repetitive sequences. Copy number alterations (CNAs) were inferred following default parameters and adjusted by tumor purity. Amplification was defined as ≥4 copies and deletion was defined as 0 copy.

Sanger sequencing

110 mutations were randomly selected for validation. Because the common detection threshold of mutations by Sanger sequencing is 10% of VAF[20], we filtered 59 mutations with a frequency of over 10%. Among these mutations, 3 mutations were excluded due to the difficulty of PCR amplification. Finally, Sanger sequencing succeed in 56 cases. 98.2% (55/56) of mutations detected by NGS were verified by Sanger sequencing (Table S2). Sequences of primers would be available upon request. Examples of Sanger sequencing validation are shown in Figure S8.

Estimation of cancer cell fraction and inference of clone status

Following the algorithm described previously[21, 22], we computed the posterior probability distribution over cancer cell fraction (CCF) of mutations to estimated their clone status. Let b denoted the number of reads supporting such mutation, d denoted the total reads covering the mutation locus, ρ referred to the tumor purity, c_t and c_n referred to the copy number of the gene locus at that base in the tumor and normal genome respectively. The expected allele-fraction $f(c)$ of a mutation present in one copy in a fraction c of cancer cells was calculated by $f(c) = c * \frac{\rho}{(1-\rho)c_n+\rho c_t}$, with $c \in [0.01,1]$. Then $P(c) \propto \text{Binomial}(b|d, f(c))$ assuming a uniform prior on c. The distribution over CCF was obtained by calculating values over a regular grid of 100 c values and normalizing. Mutations were classified as clonal on the ground of the probability that the CCF exceed

0.9. A probability threshold of 0.5 was used in our study.

To infer the proportion of tumor cells carrying a given mutation, we used the following formula[23, 24]:

$$\mathrm{CCF} = \min\left(1, \frac{b}{d} * \frac{(1-\rho)c_n + \rho c_t}{\rho}\right)$$

To test whether a gene exclusively was mutated as clonal or subclonal status, a binomial model was applied considering the ratio of clonal mutations to all mutations as the probability of success.

Classification pipeline for CCF-based pattern with influence on prognosis

Accounting for the existence of tumor heterogeneity, we hypothesized that mutations in a specific gene may have distinct impacts on patient prognosis owing to distinct cancer cell fractions of mutations. We built a classification pipeline based on the genes mutated at frequency ≥5% that integrated maximally selected rank statistics which detected optimal cutoff of biomarker on prognosis, Cox regression and log-rank test (Figure S8). R packages "survival" and "survminer" were used to calculate logrank statistics.

We first applied maximal selected rank statistics for each gene to determine the CCF cutoff that offered an optimal prediction of clinical outcomes. Then we used Cox regression and Wald test for each gene considering CCF of mutations as continuous variables to test whether mutations affected prognosis in a dose-dependent manner. For if P-value in Wald test ≤0.1, we judged the effect of mutations were continuous. These genes were classified into CCF dose-dependent manner when log-rank test between mutant and wide type was significant (P≤0.05). If P_{Wald}＞0.1 whereas $P_{Log-rank}$ between mutant and wide type was ≤0.05, and $P_{Log-rank}$ between clonal and subclonal mutations was ＞0.05, these genes were classified into CCF-independent pattern, as all mutations within a gene affected patient prognosis similarily, even those mutations with low CCF. As for those genes which $P_{Log-rank}$ between mutant and wild type was ＞0.05, then we performed log rank test based on the cutoff derived from maxstats (mutant≥ cutoff VS mutant＜ cutoff/wild type). If P-value was ≤ 0.05, then these genes were classified as CCF-dominant pattern, implying threshold effects of mutations on patient prognosis.

Ultimately, the classification was displayed and visually inspected by plotting survival curves. Through visual inspection, AHNAK was seemingly an outliner of ouralgorithms. Patients with AHNAK mutations had better OS than wild type patients. AHNAK was assigned into CCF independent pattern in the algorithm, but we still observed significant difference between clonal AHNAK mutations and subclonal mutations, indicating that the prognostic effect of AHNAK mutations actually followed a CCF-dominant pattern (Figure S7d).

Comparison of model construction using CCF data and genotype data

The CCF data were continuous and might be more informative than the discrete genotype data. To evaluate whether the use of CCF data has advantages in construction of

prognosis predictors, we used stability selection to evaluate the importance of a variable and the performance of predicting outcomes in both types of data.

Stability selection used a bootstrap-based methodology to evaluate the probability that a variable would be selected in different bootstrap-based populations. A higher probability indicated that the variable was more informative in predicting outcomes.

First, CCF and mutation status per gene per patient were assembled into a gene-sample matrix, respectively. Second, we subsampled the patients, selected variables using three popular methods (Least Absolute Shrinkage and Selection Operator, Lasso, Smoothly Clipped Absolute Deviation, SCAD and Minimax concave penalty, MCP). Finally we calculated the proportion that a variable was selected across all subsample simulations using package "hdi". Variables whose selection probabilityover 0.5 was considered as "stably selected variable". A higher proportion indicated that a variable (gene mutation) was stably selected. Then we constructed the Cox model using the most N probably selected variables, and assess the model performance.

The use of CCF data could lead to more stably selected variables compared to the use of the genotype data (Figure S6 A/B/C). In addition, the use of CCF data could achieved better model performance (Figure S6 D/E/F).

Model construction and evaluation

A predefined training and validation set were used for model construction and validation. CCF of mutations per gene per patient was assembled into a matrix. For patients without mutations in specific genes, the CCF referred to 0. The clinical endpoint we used was disease free survival (DFS). SCAD, a popular variable selection method fulfilling oracle property and providing unbiased coefficient estimation in cox proportion hazards context[25, 26], was performed (package: ncvreg) to select variables from the high dimensional matrix. The variables were further filtered by performing the stepwise Cox regression with Bayesian information criteria for the purpose of selecting independent factors significantly associated with DFS. A genetic risk score was the coefficients in the Cox model multiplying by CCF of mutations in each patient. To reach the maximal power of recurrence risk stratification, the optimal cutoff of genetic risk scores was calculated by recursive partition analysis (package: party).

We considered three scenarios for prognosis prediction: a. standard $AJCC^{8th}$ pathological stage; b. genetic variables selected above; c. standard pathological stage in a combination of genetic variables. Data of validation set was used for evaluation of model fitted in the training set. The prognostic accuracies of each variable and scenario of model were evaluated using time-dependent receiver operating characteristics (ROC) curves[27] (package: timeROC). Area under the ROC curves (AUC) were compared using Z-test[28].

To minimize the selection bias given the nature of our single center retrospective study, we further validated our predictor on the TCGA-ESCC cohort (the only available published cohort which provided omics data and date of disease progress). Only patients with detailed follow up record and pathological stage were used for validation. Briefly, the mutation profile, gene-level copy number and pathology-based tumor purity were used to calculate the cancer cell fraction of mutations as described above. Then the CCF of

mutations per gene per patient were assembled into a matrix for validation of the performance of our predictor. Several packages, including “ggplot2”, “ggsci”, “ggtheme”, “survival”, “maftools” and “trackviewer” was used for data visualization. To visualize the mutation profile of genes, the protein sequence annotations were downloaded from the Uniprot database (https://www.ebi.ac.uk/proteins/api/).

Clustering analysis of mutations using Bayesian Dirichlet Process

We employed Bayesian model-based clustering using package “DPClust” to assign mutations into subclonal cell populations. The mathematical details have been described elsewhere[24]. WES data of 96 ESCC patients in TCGA cohort was used to compare the global tumor heterogeneity in our cohort and the TCGA cohort. For both the TCGA and our cohorts, the mutation profile, gene-level copy number and tumor purity (percent of tumor nuclei calculated by pathological assessment) were processed as the input of the Bayesian Dirichlet Process.

To predict the number of subclones, the results from the clustering algorithm were processed as below[29]. Briefly, we obtained the parameters of distributions by calculating the fractions of times each number of subclones inferred by the Bayesian Dirichlet process. For those subclones that harbor more than one mutation, have expected CCF ≥0.1, with sample purity ≥0.7, we simulated 2000 trials under the distributions above and calculated the total number of subclones observed in patients in each draw. To generate Fig 1c, we calculated the mean and standard deviation across the trials.

SUPPLEMENTARY FIGURE

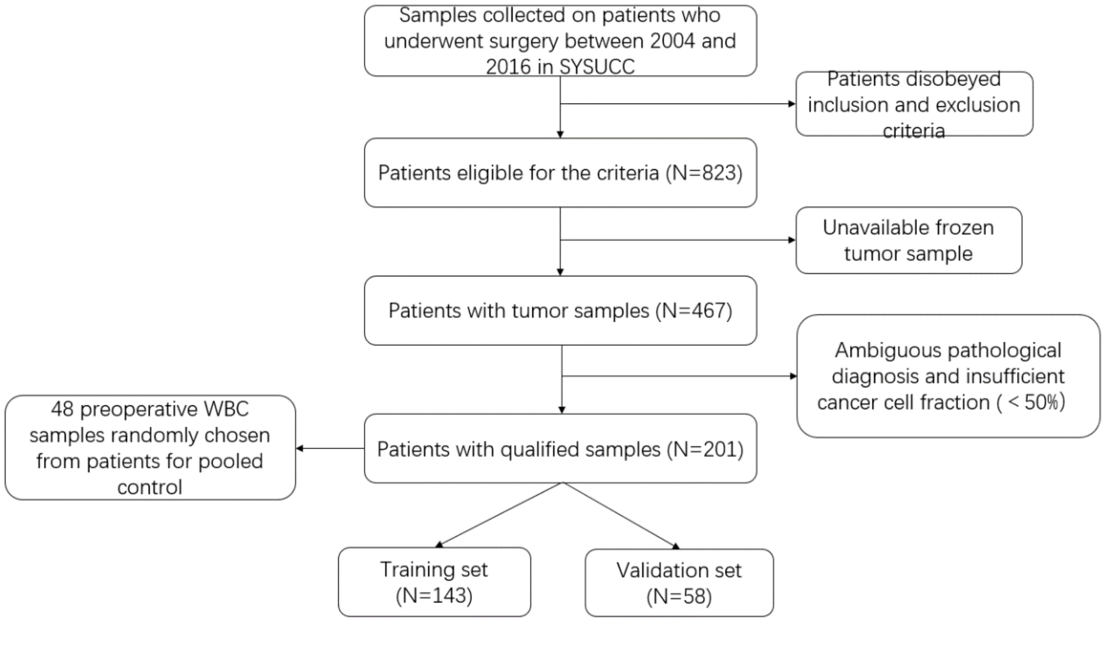

Figure S1. Diagram of sample selection.

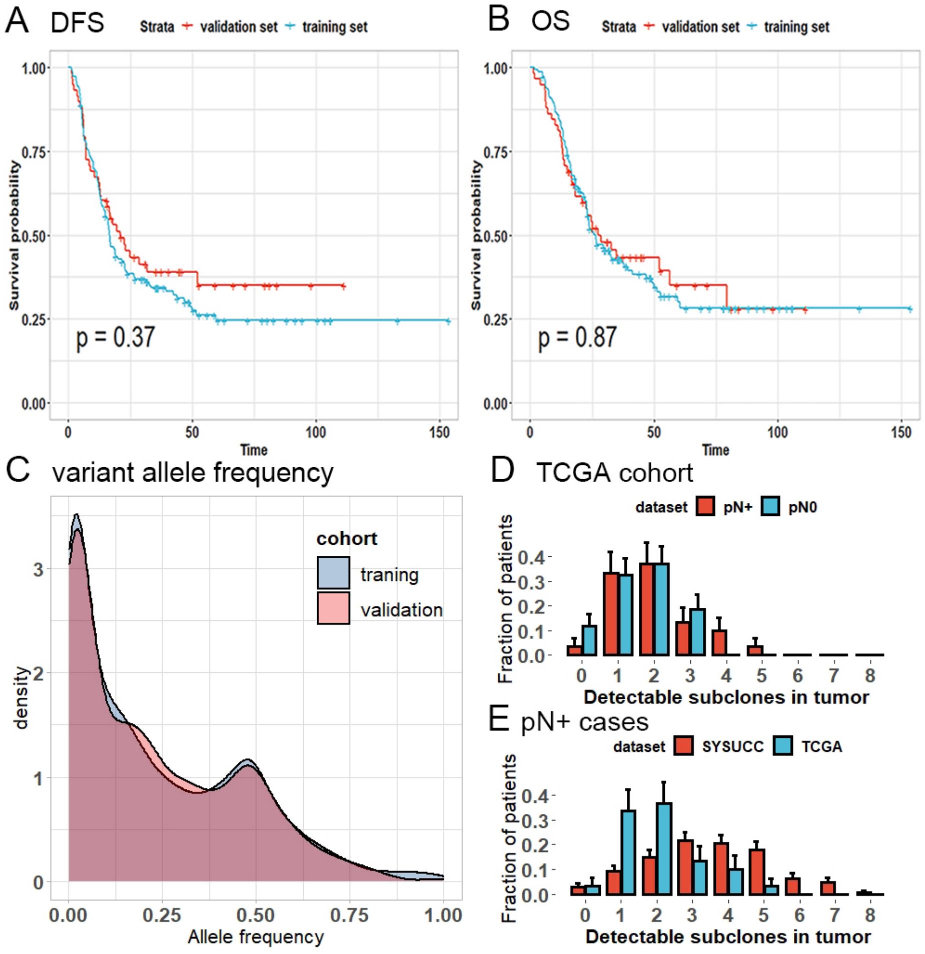

Figure S2. Comparison between training cohort and validation cohort. (A/B) Survival curves of DFS (**A**) and OS (**B**) between training and validation set. P value was calculated with log rank test. (**C**) Distribution of allele frequency of mutations in training and validation cohort. (**D**) Predicted number of subclones in pN0 and pN+ ESCC in TCGA dataset. (**E**) Predicted number of subclones in pN+ ESCC in our cohort and TCGA dataset.

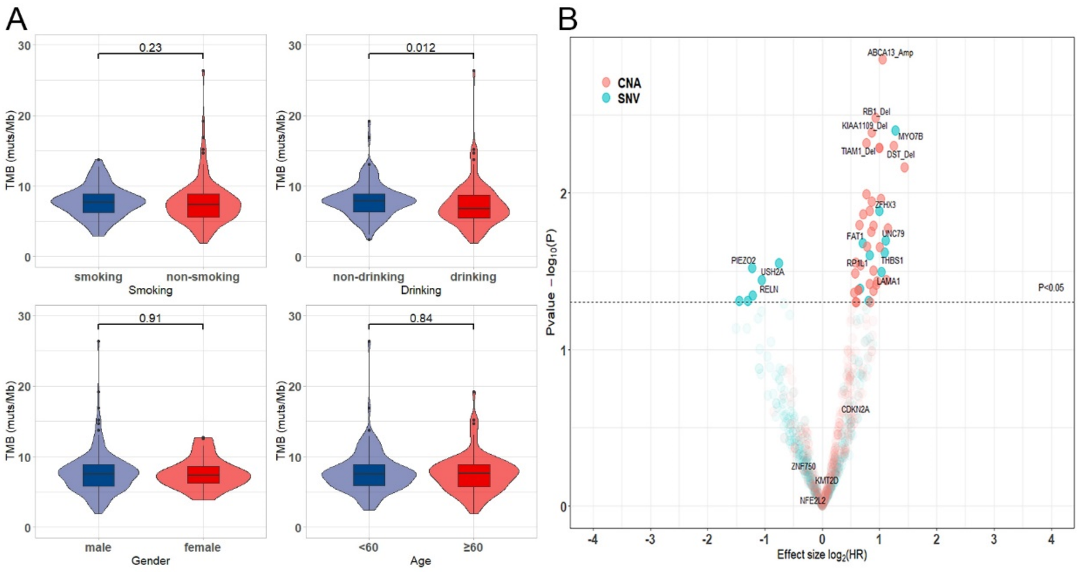

Figure S3. Clinical relevance of genetic features. (**a**) Correlation between tumor mutation burden (TMB) and clinical characteristics. P value was calculated with wilcoxon rank sum test. (**b**) Volcano plot displayed the relationship between genetic alterations and OS. The X and Y axes indicated the log_2^{HR} and $-log_{10}^{P}$, respectively.

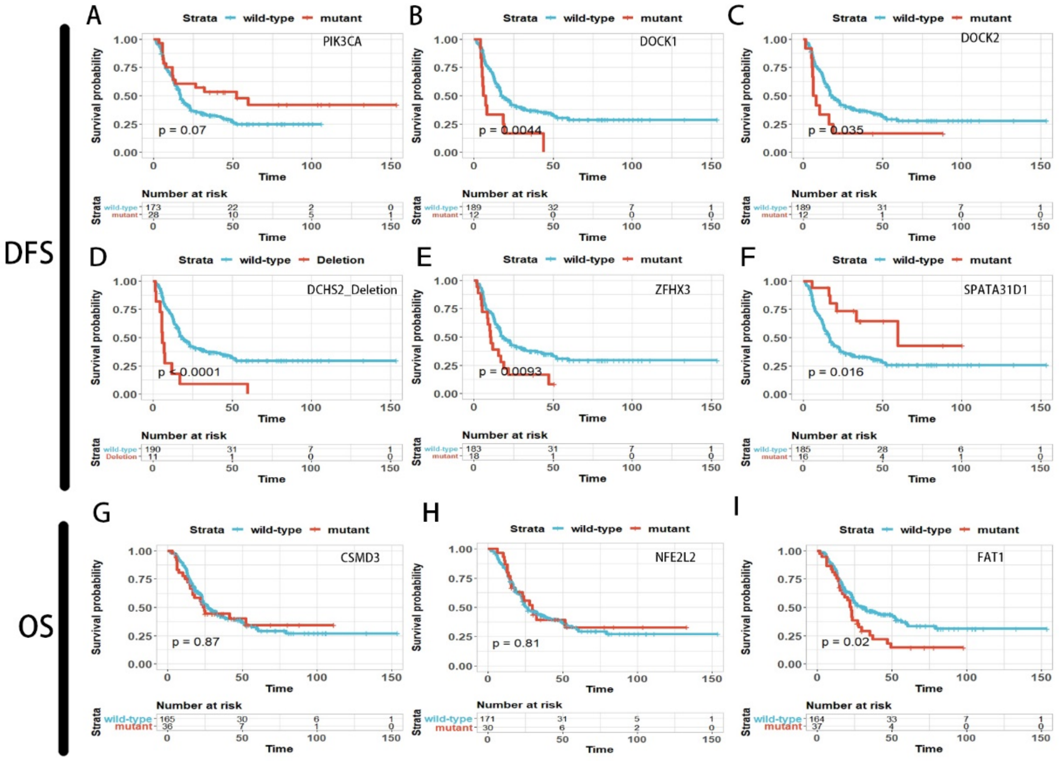

Figure S4. Survival curves of patients grouped by mutation status of genes. P values were calculated with log rank test.

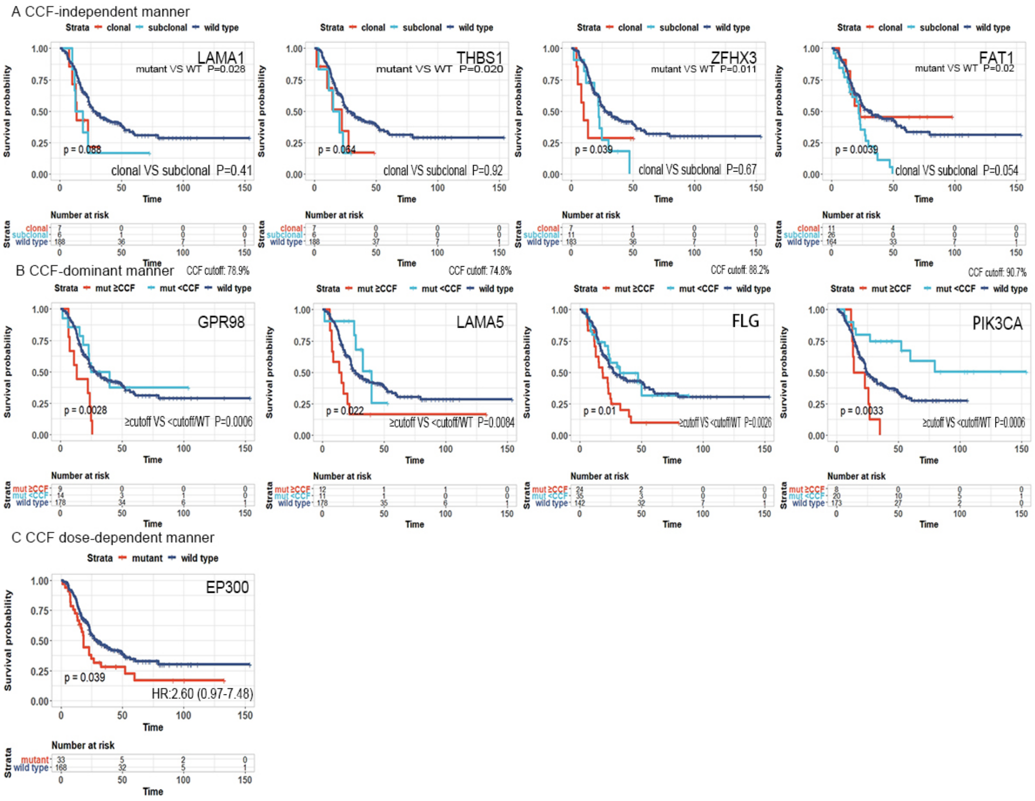

Figure S5. CCF-based patterns of prognostic value. The clinical endpoint analyzed here was OS. Prognostic effect of mutations was classified into three patterns according to our algorithm: CCF-independent pattern (A), CCF-dominant pattern (B) and CCF dose-dependent pattern (C). HR was calculated using Cox model.

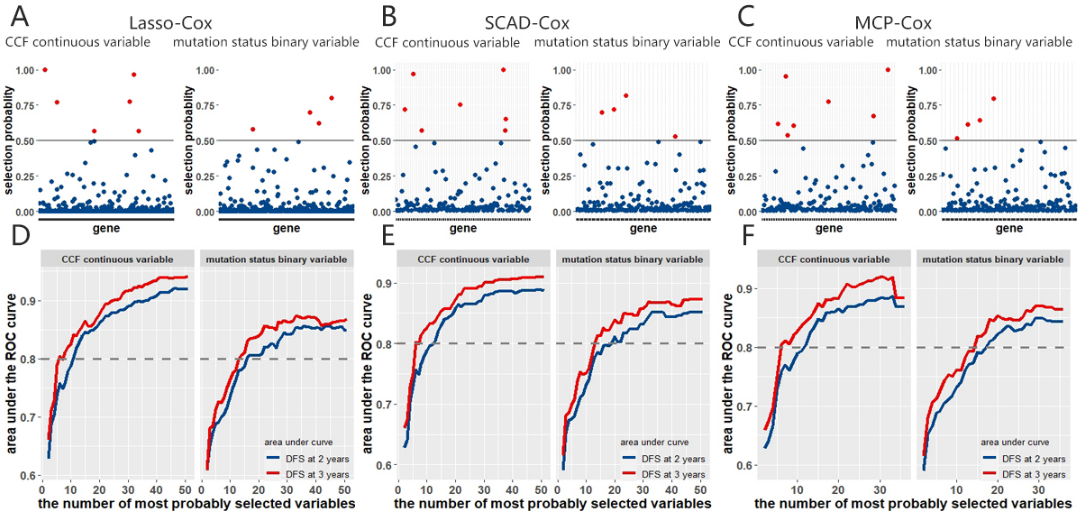

Figure S6. Variable selection based on CCF and mutation status data. Variable selection and model construction were performed using the CCF and mutation status data. (A/B/C) scatter plot displaying the probablity of variables selected by three different methods, Lasso (A), SCAD (B) and MCP (C). "Stably selected variables" were dotted in red and unstable selected variables were dotted in blue. Using CCF data yielded a higher number of stably selected variables than using mutation status data. (D/E/F) Prediction accuracy of the model using the most N probably selected variables. Using CCF data could achieve higher AUCs with fewer variables than using the mutation status data.

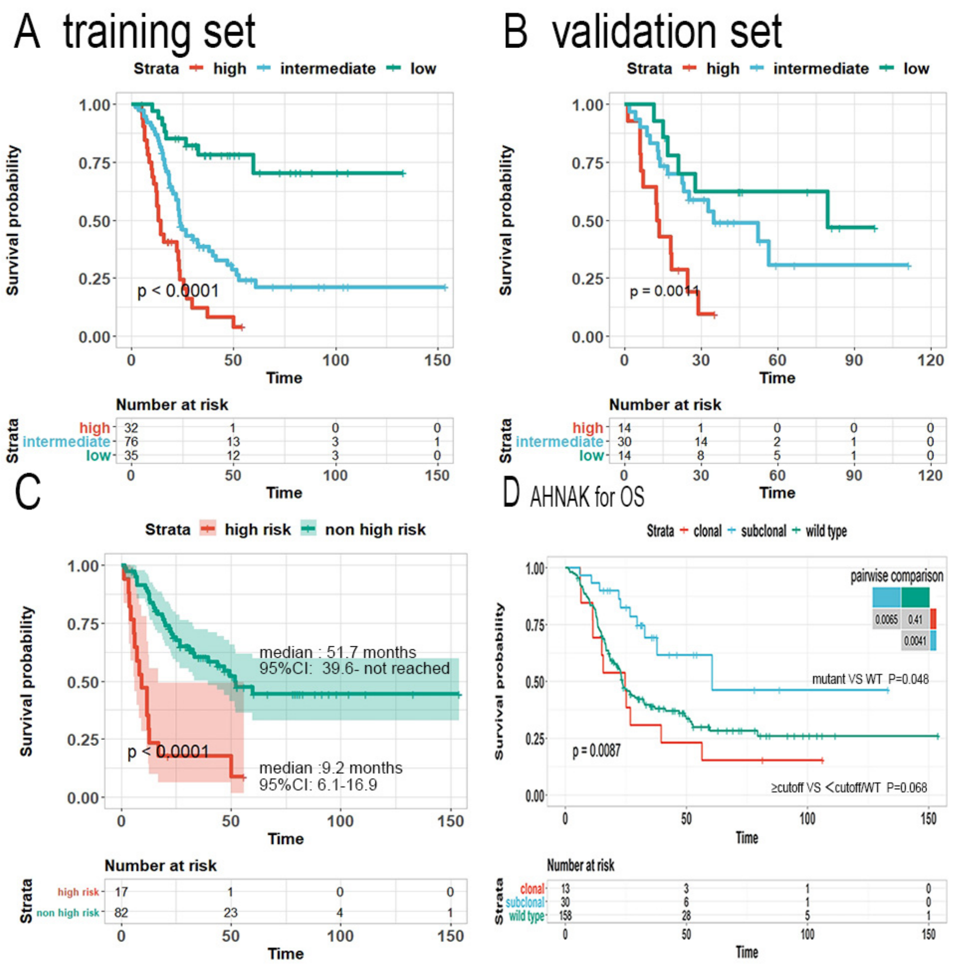

Figure S7. Kaplan-Meier Curves for OS of patients with distinct recurrence risks. (A/B) Patients with different recurrence risks also had distinct overall survival patterns in both the training set (**A**) validation set (**B**). (**C**) Survival curves of N_1 patients stratified into two groups, high risk group and non high risk group. (**D**) Survival curves of patients grouping by different mutation status of AHNAK.

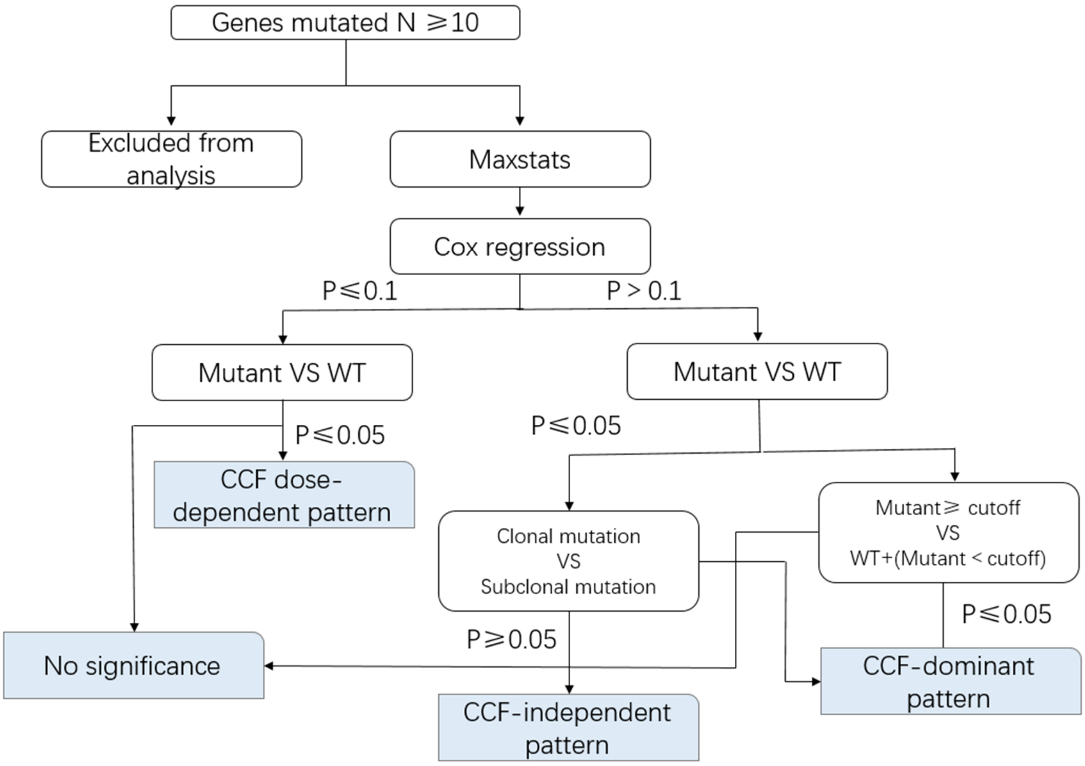

Figure S8. Classification pipeline for distinguishing prognosis effect patterns of mutations. The pipeline was constructed using Cox regression, log-rank test and maximal selected rank statistics.

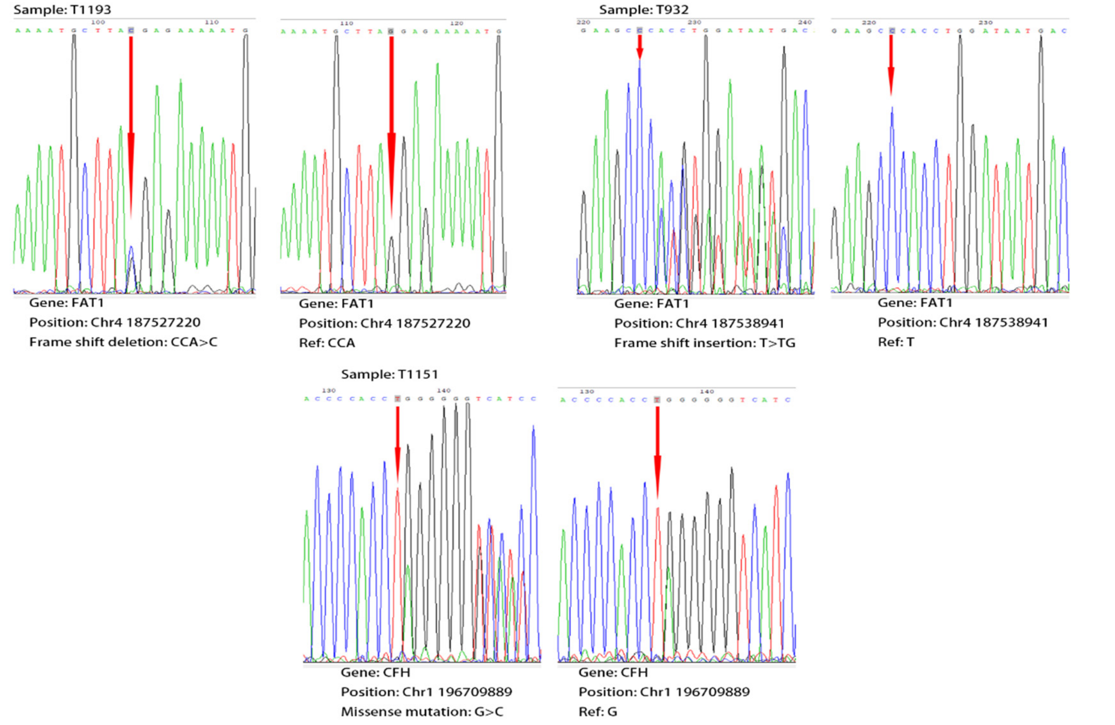

Figure S9. Examples of Sanger sequencing validation. The germline variant presented in T932 was filtered by our bespoke pipeline.

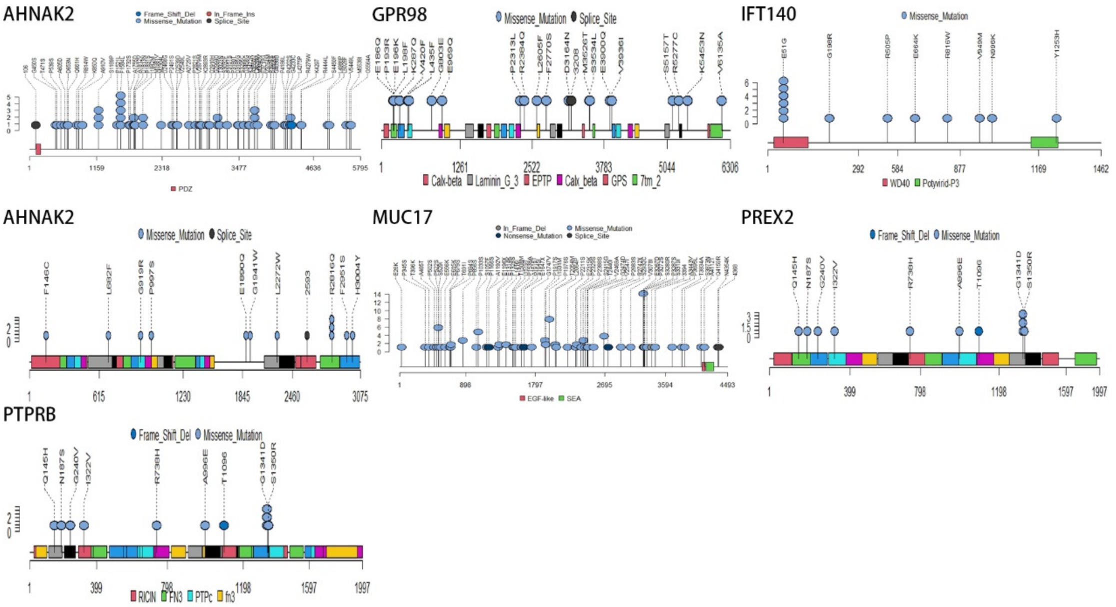

Figure S10. Lillipop figures displaying distribution of mutations of the eight genes included in the recurrence predictors.

Table S1. Summarization of studies used for designing our gene panel. EAC and ESCC are the abbreviation of esophageal adenocarcinoma and squamous cell carcinoma.

**Author**	**Year**	**Samples**	**Geographic origin**	**Sequencing technique**	**Median Depth**
Agrawal, N., et al.	2012	EAC(11 cases) and ESCC(12 cases)	Maryland, America	WES	157X
Gao, Y. B., et al.	2014	ESCC with paired normal tissues	Beijing,China	WES(113 cases)	122X
Lin, D. C., et al.	2014	ESCC with paired normal tissues	Beijing,China	WGS (20 cases)	79X
				WES (119 cases)	111X
Song, Y., et al.	2014	ESCC with paired normal tissues	Chaoshan high incidence area of ESCC,China	WGS (17 cases)	> 30X
				WES (141 cases)	> 100X
Zhang, L., et al.	2015	ESCC with paired normal tissues	Taihang Mountains high incidence area of ESCC,China	WGS (14 cases)	65X
				WES (90 cases)	132X
Qin, H. D., et al.	2016	ESCC with paired normal tissues	Guang zhou,China	WGS (10 cases)	70X
				WES (60 cases)	
Sawada, G., et al	2016	ESCC with paired normal tissues	Japan	WES(144 cases)	120X
Dai, W., et al.	2017	ESCC samples	Hong Kong,China	WES(cases)	80X

Table S3. Clinical variables associated with gene mutations. P value are calculated with Fisher's exact test with Bonferroni-Holm correction for multiple comparison.

Clinical variables	Gene	Mutation frequency(VS no risk factor)	FDR value
smoking	EP300	22.4% (27/129) VS 9.7% (6/72)	0.028
	CASZ1	7.0%(9/129) VS 0%	0.028
	MYH4	11.6% (15/129) VS 2.8% (2/72)	0.034
drinking	FAT1	25.3% (25/99) VS 11.8% (12/102)	0.018
	ADAM29	7.1% (7/99) VS 0.9% (1/101)	0.033
	EP300	22% (22/99) VS 10.8% (11/102)	0.036
age >60 years	TET2	14.7% (16/109) VS 4.3% (4/92)	0.017
	FBXW7	2.1% (2/92) VS 11.0% (12/109)	0.023
	CREBBP	8.7% (8/92) VS 19.3% (21/109)	0.043
pN2-3	MYO7B	11.8% (12/102) VS 3.0% (3/99)	0.029
	DNAH9	15.7% (16/102) VS 6.0% (6/99)	0.041
female	LOXHD1	17.1% (6/35) VS 3.6% (6/166)	0.007
	ABCC9	14.3% (5/35) VS 3.0% (5/166)	0.016

Table S8. Probability of the right genes being selected into the CCF-based predictor during 1000 simulations.

Gene	Lasso-Cox (%)	SCAD-Cox (%)	MCP-Cox (%)
GPR98	48.5	48	39.5
LAMA1	19	19.5	26.5
IFT140	12.5	14	15.5
MUC17	15	18.5	18.5
PTPRB	40	45.5	53.5
AHNAK	100	100	100
PREX2	96.5	97	95
SPATA31D1	43	48	44

1 Liu J, Lichtenberg T, Hoadley KA, et al. An Integrated TCGA Pan-Cancer Clinical Data Resource to Drive High-Quality Survival Outcome Analytics. *Cell*. 2018; 173: 400-416 e411.

2 Dai W, Ko JMY, Choi SSA, et al. Whole-exome sequencing reveals critical genes underlying metastasis in oesophageal squamous cell carcinoma. *J Pathol*. 2017; 242: 500-510.

3 Sawada G, Niida A, Uchi R, et al. Genomic Landscape of Esophageal Squamous Cell Carcinoma in a Japanese Population. *Gastroenterology*. 2016; 150: 1171-1182.

4 Qin HD, Liao XY, Chen YB, et al. Genomic Characterization of Esophageal Squamous Cell Carcinoma Reveals Critical Genes Underlying Tumorigenesis and Poor Prognosis. *Am J Hum Genet*. 2016; 98: 709-727.

5 Zhang L, Zhou Y, Cheng C, et al. Genomic analyses reveal mutational signatures and frequently altered genes in esophageal squamous cell carcinoma. *Am J Hum Genet*. 2015; 96: 597-611.

6 Song Y, Li L, Ou Y, et al. Identification of genomic alterations in oesophageal squamous

cell cancer. *Nature*. 2014; 509: 91-95.

7 Lin DC, Hao JJ, Nagata Y, et al. Genomic and molecular characterization of esophageal squamous cell carcinoma. *Nat Genet*. 2014; 46: 467-473.

8 Gao YB, Chen ZL, Li JG, et al. Genetic landscape of esophageal squamous cell carcinoma. *Nat Genet*. 2014; 46: 1097-1102.

9 Agrawal N, Jiao Y, Bettegowda C, et al. Comparative genomic analysis of esophageal adenocarcinoma and squamous cell carcinoma. *Cancer Discov*. 2012; 2: 899-905.

10 Li H, Durbin R. Fast and accurate short read alignment with Burrows-Wheeler transform. *Bioinformatics*. 2009; 25: 1754-1760.

11 Tarasov A, Vilella AJ, Cuppen E, Nijman IJ, Prins P. Sambamba: fast processing of NGS alignment formats. *Bioinformatics*. 2015; 31: 2032-2034.

12 Cibulskis K, Lawrence MS, Carter SL, et al. Sensitive detection of somatic point mutations in impure and heterogeneous cancer samples. *Nat Biotechnol*. 2013; 31: 213-219.

13 Wang K, Li M, Hakonarson H. ANNOVAR: functional annotation of genetic variants from high-throughput sequencing data. *Nucleic Acids Res*. 2010; 38: e164.

14 Stuart E. Lacy, * Sharon L. Barrans,2, Paul Glover,2, Simon Crouch, † * Philip A. Beer,3, * Daniel Painter,1, Suzan J. L. Van Hoppe NW, 2 and Daniel J. Hodson7. Targeted sequencing in DLBCL, molecular subtypes, and outcomes: a Haematological Malignancy Research Network report. *Blood*.

15 Papaemmanuil E, Gerstung M, Bullinger L, et al. Genomic Classification and Prognosis in Acute Myeloid Leukemia. *N Engl J Med*. 2016; 374: 2209-2221.

16 Papaemmanuil E, Gerstung M, Malcovati L, et al. Clinical and biological implications of driver mutations in myelodysplastic syndromes. *Blood*. 2013; 122: 3616-3627; quiz 3699.

17 Forbes SA, Bindal N, Bamford S, et al. COSMIC: mining complete cancer genomes in the Catalogue of Somatic Mutations in Cancer. *Nucleic Acids Res*. 2011; 39: D945-950.

18 Lek M, Karczewski KJ, Minikel EV, et al. Analysis of protein-coding genetic variation in 60,706 humans. *Nature*. 2016; 536: 285-291.

19 Talevich E, Shain AH, Botton T, Bastian BC. CNVkit: Genome-Wide Copy Number Detection and Visualization from Targeted DNA Sequencing. *PLoS Comput Biol*. 2016; 12: e1004873.

20 Nadeu F, Delgado J, Royo C, et al. Clinical impact of clonal and subclonal TP53, SF3B1, BIRC3, NOTCH1, and ATM mutations in chronic lymphocytic leukemia. *Blood*. 2016; 127: 2122-2130.

21 Landau DA, Carter SL, Stojanov P, et al. Evolution and impact of subclonal mutations in chronic lymphocytic leukemia. *Cell*. 2013; 152: 714-726.

22 Landau DA, Tausch E, Taylor-Weiner AN, et al. Mutations driving CLL and their evolution in progression and relapse. *Nature*. 2015; 526: 525-530.

23 Dentr SC. Principles of reconstructing the subclonal architecture of cancers.

24 Nik-Zainal S, Van Loo P, Wedge DC, et al. The life history of 21 breast cancers. *Cell*. 2012; 149: 994-1007.

25 Breheny P, Huang J. COORDINATE DESCENT ALGORITHMS FOR NONCONVEX PENALIZED REGRESSION, WITH APPLICATIONS TO BIOLOGICAL FEATURE

SELECTION. *The annals of applied statistics*. 2011; 5: 232-253.

26 Fan J, Li R. Variable Selection via Nonconcave Penalized Likelihood and its Oracle Properties. *Journal of the American Statistical Association*. 2001; 96: 1348-1360.

27 Blanche P, Dartigues JF, Jacqmin-Gadda H. Estimating and comparing time-dependent areas under receiver operating characteristic curves for censored event times with competing risks. *Stat Med*. 2013; 32: 5381-5397.

28 Robin X, Turck N, Hainard A, et al. pROC: an open-source package for R and S+ to analyze and compare ROC curves. *BMC Bioinformatics*. 2011; 12: 77.

29 Lohr JG. Widespread genetic heterogeneity in multiple myeloma: implications for targeted therapy. *Cancer Cell*.
